# 2145. Antagonistic Activity of *Lacticseibacillus rhamnosus* R3 Against Carbapenem-Resistant *Acinetobacter baumannii* Strain: A Promising Therapeutic Alternative

**DOI:** 10.1093/ofid/ofad500.1768

**Published:** 2023-11-27

**Authors:** Cecilia Rodriguez, Dema Ramlaoui, Nardin Georgeos, Briea Gasca, Camila Leal, Nicholas Salzameda, Robert A Bonomo, Raul Raya, Maria Soledad Ramirez

**Affiliations:** Centro de Referencia para Lactobacilos (CERELA-CONICET), Tucuman, Tucuman, Argentina; CSUF, Fullerton, California; CSUF, Fullerton, California; CSUF, Fullerton, California; CERELA, Tucuman, Tucuman, Argentina; CSUF, Fullerton, California; Louis Stokes Cleveland Department of Veterans Affairs Medical Center, Cleveland,, Cleveland, Ohio; CERELA, Tucuman, Tucuman, Argentina; California State University Fullerton, Corona, California

## Abstract

**Background:**

*Acinetobacter baumannii (*AB) is a recognized nosocomial pathogen of critical importance Most circulating AB strains are carbapenem-resistant (CRAB), which severely complicates therapeutics with available antibiotics. Probiotic lactic acid bacteria constitute a promising therapeutic alternative. Previously, we determined that *Lacticaseibacillus rhamnosus* R3 (LR-R3) exerts a strong inhibitory capacity on the model strain A118 (susceptible to antibiotics). In this analysis, the antimicrobial activity of LR-R3 against type CRAB hypervirulent strain AB5075 was evaluated.

Figure 1

**Figure:**
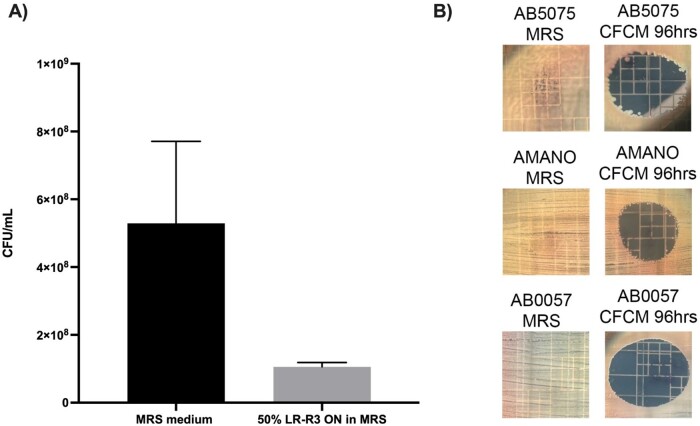

A) Transwell migration assay. AB5075 viability after 24h exposure to LR-R3 CFCM. B) CFCM activity obtained from 96h culture of LR-R3 on CRAB strains.

**Methods:**

The inhibitory activity of cell pellet and its cell-free supernatants (CFCM) were determined, respectively, by the soft-agar overlay method, CFCM activity, and transwell migration assay. Also, killing assays and scanning microscopy imaging were performed under co-culture conditions. Lastly, AB5075 transcriptional response when exposed to LR-R3 was assessed by RT-qPCR.

**Results:**

The lactic acid bacteria LR-R3 demonstrated a strong inhibitory capacity on AB5075 strain (inhibition halo diameter, DHI >20 mm), with loss of cell viability at 4h of co-culture conditions. The CFCM of LR-R3 also showed similar antimicrobial activity on AB5075 (Fig 1A) and against others CRAB strains (Fig 1B), suggesting that the inhibitory effect observed is due to the presence of a compound or metabolite produced and secreted by the lactobacillus.

Microscopy images showed increase formation of outer membrane vesicles (OMVs) and the presence of nanotubes. In addition, significant changes in the expression levels of the studied genes were observed in AB5075 in co-culture with LR-R3: increase in the expression of genes involved in biofilm (*csuAB*, *csuB*, *ompA*); and decrease in the expression of genes involved in the synthesis and utilization of iron (*bauA*, *exbD*, *bfnA*, *basE, pirA*), and genes related to fatty acid and lipid metabolism (*paaA*, *paaB*).

**Conclusion:**

The results obtained allowed the identification of *Lcb. rhamnosus* R3 as a probiotic strain with antagonistic activity on the nosocomial pathogen *A. baumannii*. In addition, it was shown that LR-R3 alters iron and fatty acid metabolism, by virtue of this interaction. This work constitutes the basis for future studies necessary to elucidate the mechanism involved.

**Disclosures:**

**Robert A. bonomo, MD**, Entasis, Merck, VenatoRx, Wockhardt: Grant/Research Support

